# Benchmarking Excellence: Evaluating Advanced Breast Carcinoma Care in Pakistan's Largest Cancer Hospital Against the National Institute for Health and Care Excellence (NICE) Guidelines

**DOI:** 10.7759/cureus.44332

**Published:** 2023-08-29

**Authors:** Muhammad Awais Kanwal, Umaisa Khalid, Momina Amir, Barka Sajjad, Rana Zeeshan, Namra Urooj, Nifasat Farooqi, Muhammad Asad Parvaiz, Amina Iqbal Khan, Mohammad Zulqarnain Chaudhry

**Affiliations:** 1 Surgical Oncology, Shaukat Khanum Memorial Cancer Hospital and Research Centre, Lahore, PAK

**Keywords:** breast cancer, palliative care, general surgery, nice guidelines, oncology, breast surgery, quality improvement project, clinical audit, advanced breast carcinoma, cancer

## Abstract

Introduction

Breast cancer is the most common type of cancer worldwide, and even with all the screening and education, great numbers of diagnoses are made in advanced stages. Additionally, patients in remission always remain at risk of relapse and metastasis. Pakistan has the highest incidence of breast cancer among Asian countries. The purpose of this clinical audit was to compare data from the largest cancer hospital in Pakistan with international standards to provide room for quality improvement.

Methods

A retrospective review of patients with advanced breast carcinoma over a period of six months was done. Permission was obtained from the Quality Assurance and Patent Safety Department before the commencement of the audit.

Standards

Data obtained were audited against nine standards of four different categories from the National Institute for Health and Care Excellence (NICE) guidelines on advanced breast carcinoma.

Results

For the diagnosis and assessment category, for which a target of 100% was set, 99.66% was achieved; for disease monitoring, for which a target of 100% was set, 91.8% was achieved; for systemic disease-modifying therapy, for which the majority was the target, only 1% was achieved; for managing complications, for which a target of 100% was set, 71.8% was achieved.

Conclusion

Continuous research and breakthrough advancements have made health care an ever-evolving field. Clinical audits like these that compare international standards with local data are beneficial and lead to quality improvement. They highlight issues that may be overlooked otherwise, raise questions that may never be asked, and may inspire prospective research studies. Limitations of the audit were that this clinical audit was conducted outside of the NHS where NICE guidelines are not followed and local guidelines differ from NICE guidelines.

## Introduction

Breast cancer is the most common malignancy in women worldwide. Metastasis is the leading cause of high mortality in most cancers [1]. Advanced or metastatic breast carcinoma is a frightening diagnosis. Breast carcinoma, even if diagnosed earlier and treated promptly, may metastasize, leading to poor quality of life. Caring for patients with advanced stages of breast carcinoma is quite challenging and needs specialized healthcare facilities. It is possible to provide a good quality of life to patients with advanced stages of breast carcinoma with well-planned individualized treatment plans. Finding support for coping with everyday life and dealing with feelings of anxiety and grief is particularly important [2].

With advanced breast carcinoma, the focus is on limiting the growth of the tumor, delaying the progress of the disease, and relieving the symptoms. Individualized treatment plans are based on the type of primary tumor, number and site of secondary metastasis, biological characteristics of the tumor, age, physical condition, and wishes of the patient after providing adequate information, and support for decisions is an important consideration.

Uncertainty in terms of disease progression and life expectancy makes advanced breast carcinoma a difficult disease to work with. Despite the seriousness of the disease, there is a good number of patients who live a good quality life with individualized care plans. According to the Munich Cancer Registry, about 23 out of 100 women are still alive five years after they are diagnosed with breast cancer with distant metastases. This is the case for 13 out of 100 women after 10 years [2].

Controlling local disease, managing nutrition, providing adequate pain relief, and managing complications like lymphedema and cancer-related fatigue are the mainstay of treatment. Clinical audits and quality improvement projects have proven to be effective in driving quality improvement initiatives within healthcare facilities. By systematically assessing the current practices and comparing them with established standards, clinical audits can identify gaps and areas for improvement. These audits provide valuable insights into the strengths and weaknesses of the healthcare system, enabling targeted interventions and enhancements in patient care [3].

Breast cancer is a significant health concern, and evaluating the quality of care provided to patients with advanced breast carcinoma is crucial. It is essential to ensure that the care provided aligns with international best practices and guidelines, such as those set by the National Institute for Health and Care Excellence (NICE).

The NICE guidelines are evidence-based recommendations that provide a benchmark for high-quality breast carcinoma care. They encompass various aspects, including diagnosis, treatment, supportive care, and survivorship [4].

In this clinical audit, we determined areas where improvements can be made to enhance patient outcomes and overall healthcare delivery. This was done by systematically reviewing medical records, treatment protocols, and patient outcomes. This provided valuable data on the hospital's strengths and weaknesses, allowing for targeted interventions and the implementation of evidence-based changes. By highlighting areas of improvement and sharing the findings, we promote a culture of continuous learning and quality improvement within the healthcare community.

## Materials and methods

A retrospective review of electronic health records of patients with advanced breast carcinoma over a period of six months was done. Different standards from NICE guidelines for advanced breast cancer diagnosis and treatment were audited against local practices.

Approval

Permission was obtained from the Quality and Patient Safety Department of Shaukat Khanum Memorial Cancer Hospital and Research Centre, Lahore before the commencement of the audit.

Statistical methods

The retrospective data collected were analyzed using Statistical Package for Social Sciences version 20 (IBM Corp., Armonk, NY). Categorical variables were reported as numbers and percentages. The Mann-Whitney U test was used to compare differences in continuous variables. Values of categorical variables were compared by the chi-square test. A p-value of <0.05 was considered significant.

## Results

The diagnosis and assessment category is the most important part of cancer management. Three standards from the NICE guidelines on advanced breast carcinoma were compared with local data, for which a target of 100% was set, and a cumulative score of 99.66% was achieved.

Disease monitoring is very important when relapse and metastatic breast cancer are in discussion. One standard was compared for which a target of 100% was set, and 91.8% was achieved.

For systemic disease-modifying therapy, for which the NICE guidelines advise a target of "Majority," only 1% was achieved.

Four standards from the category of managing complications were compared, for which a target of 100% was set and a cumulative score of 71.8% was achieved. Details are tabulated below in Table 1.

**Table 1:** Results Data were obtained after searching the hospital information system (HIS) for patients being treated with advanced breast carcinoma in the last six months for the first cycle of this clinical audit. A total of 78 patients were identified with advanced breast carcinoma and electronic health records (EHR) of these patients were assessed and audited against the nine identified standards from the National Institute for Health and Care Excellence (NICE) guidelines on advanced breast carcinoma. Audit standards are taken from the NICE guidelines on advanced breast cancer: diagnosis and treatment.

Audit standards	Category	NICE reference	Guideline	Questions used for data collection	Exceptions	Target	Achieved
Audit Standard 1	Diagnosis & assessment	1.1.1	“Assess the presence and extent of visceral metastases using a combination of plain radiography, ultrasound, computed tomography (CT) scans, and magnetic resonance imaging (MRI).”	Q #1. Was adequate metastatic work-up done for staging? Q #2. Which tests were done?	None	100%	100%
Audit Standard 2	Diagnosis & assessment	1.1.5	“Positron emission tomography fused with computed tomography (PET-CT) should only be used to make a new diagnosis of metastases for patients with breast cancer whose imaging is suspicious but not diagnostic of metastatic disease.”	Q #1. Was the patient’s imaging suspicious but not diagnostic of metastatic disease? Q #2. Was PET-CT used to make a new diagnosis of metastases?	None	100%	99%
Audit Standard 3	Diagnosis & assessment	1.1.6	“On recurrence, consider reassessing estrogen receptor (ER) and human epidermal growth factor 2 receptor (HER2) status if a change in receptor status will lead to a change in management.”	Q #1. Was the receptor status assessed at the time of initial diagnosis? Q #2. Was there any local re-occurrence, mention date if any? Q #3. Was the receptor status assessed at the time of disease recurrence? Q #4. What was the patient’s initial receptor status? Q #5. What was the patient’s receptor status on recurrence?	When no tissue is available and further biopsy is not feasible or declined	100%	100%
Audit Standard 4	Disease monitoring	1.1.8	“Do not use PET-CT to monitor advanced breast cancer.”	Q #1. Was PET-CT done more than once? Q #2. Was PET-CT done for re-staging scans?	None	100%	91.8%
Audit Standard 5	Systemic disease-modifying therapy	1.3.1	“Offer endocrine therapy as first-line treatment for the majority of patients with ER-positive advanced breast cancer.”	Q #1. Was the receptor status assessed at the time of initial diagnosis? Q #2. What was the patient’s initial receptor status? Q #3. If the patient was ER-positive, were they offered endocrine therapy as the first-line treatment?	The patient has ER-positive advanced breast cancer, which is imminently life-threatening or requires early relief of symptoms because of significant visceral organ involvement, provided they understand and are prepared to accept toxicity. The patient is already being treated with chemotherapy	Majority	1%
Audit Standard 6	Managing complications	1.5.19	“Where external beam radiotherapy is used to treat patients with bone metastases and pain, this should be done in a single fraction of 8Gy.”	Q #1. Was external beam radiotherapy offered to patients with bone metastasis? Q #2. Were there other potential or current bone complications? Q #3. Please state doses if bone radiotherapy was done Q #4. Number of fractions?	Where treatment of other potential or current bone complications requires longer fractionation	100%	41.6%
Audit Standard 7	Managing complications	1.5.2	“Discuss with people who have or who are at risk of breast cancer-related lymphedema that exercise may improve their quality of life.”	Q #1. Patients with a risk of lymphedema (after axillary clearance surgery) seen by a physiotherapist?	None	100%	74%
Audit Standard 8	Managing complications	1.5.19	“Offer whole brain radiotherapy to patients for whom surgery is not appropriate unless they have a very poor prognosis.”	Q #1. In the case of brain metastases, was the brain metastases pathway followed? Q #2. Reviewed by neurosurgeon? Q #3. Reviewed by the radiation oncology team? Q #4. Brain radiotherapy?	None	100%	100%
Audit Standard 9	Managing complications	1.5.11	“A breast cancer multidisciplinary team should assess all patients presenting with uncontrolled local disease and discuss the therapeutic options for controlling the disease and relieving symptoms.”	Q #1 Was the patient assessed by a multidisciplinary team?	None	100%	100%

## Discussion

Diagnosis and assessment is the most important part of a multidisciplinary approach to dealing with cancer. Visceral metastases refer to the spread of breast cancer cells to other organs in the body, such as the lungs, liver, or bones. Staging of the disease is very important, as it has profound effects on management and prognosis. Imaging for staging needs to be targeted toward those most likely to have disseminated disease, followed by multidisciplinary discussion about various modalities of treatment that can be offered [5].

Bone scans are sensitive in detecting bone metastases, even before they become visible on other imaging modalities like plain radiography or CT scans. They can help identify the presence, location, and extent of bone involvement in advanced breast carcinoma, aiding in treatment planning and monitoring disease progression [6].

Metastatic cancer, characterized by the spread of cancer cells from the primary site to distant organs, poses a significant challenge in terms of accurate staging and subsequent treatment planning. Various imaging modalities, such as CT, MRI, and positron emission tomography fused with computed tomography (PET-CT), play crucial roles in diagnosing and staging metastatic cancer. However, the use of these imaging techniques should be judiciously employed to balance the need for accurate staging with the potential drawbacks of increased costs and radiation burden, considering the overall low metastatic results, the potential drawbacks associated with excessive imaging, and the prudent utilization of PET-CT scans.

Metastasis, though a serious complication of cancer, occurs relatively infrequently in the overall patient population. Consequently, subjecting every cancer patient to extensive imaging with CT scans and MRIs may lead to unnecessary costs and radiation exposure. The decision to stage a patient for metastasis should be based on clinical factors, including the primary cancer type, tumor characteristics, and the presence of symptoms suggestive of metastasis. This targeted approach ensures that imaging resources are utilized efficiently, minimizing the potential drawbacks associated with widespread imaging.

Performing CT scans and MRIs on all cancer patients, irrespective of their metastatic risk, would significantly escalate healthcare costs. These imaging modalities involve high expenses in terms of equipment, personnel, and interpretation. Moreover, excessive use of ionizing radiation in CT scans poses a potential risk of radiation-induced secondary malignancies. Balancing the need for accurate staging with the economic and health burden imposed by imaging procedures is essential for optimal patient care.

PET-CT is a valuable tool in cancer imaging, combining the functional information provided by PET with the anatomical details obtained from CT scans. However, due to its high cost and exposure to radiation, PET-CT should be employed judiciously. It should be reserved for cases where other imaging modalities, such as CT and MRI, fail to provide conclusive results. PET-CT can offer valuable insights into the metabolic activity of lesions, aiding in differentiating benign from malignant findings and guiding treatment decisions.

In the staging of metastatic cancer, it is crucial to strike a balance between the need for accurate assessment and the potential drawbacks associated with extensive imaging. Given the overall low metastatic results, it is not cost-effective or beneficial to subject all patients to CT scans and MRIs. Instead, clinical factors should guide the decision to perform imaging, ensuring optimal utilization of resources. Similarly, PET-CT should be reserved for cases where other imaging modalities are inconclusive, thus maximizing its benefits while minimizing costs and radiation burden. By adopting a selective approach to imaging, healthcare providers can optimize the staging process and enhance patient outcomes.

PET-CT combines two imaging techniques, i.e., positron emission tomography (PET) and computed tomography (CT). PET detects metabolic changes by using a radioactive tracer, while CT provides anatomical details. This fusion imaging modality has gained popularity in cancer diagnosis and staging. However, when it comes to monitoring advanced breast cancer, several limitations must be acknowledged.

PET-CT involves the use of ionizing radiation, which poses potential risks, especially when repeated scans are required for monitoring purposes. The cumulative radiation exposure can be a concern, particularly in younger patients or those requiring long-term monitoring. Additionally, PET-CT is a costly imaging modality, which can impose a financial burden on patients and healthcare systems. Utilizing PET-CT for routine monitoring may not be feasible or cost-effective, considering these limitations.

Estrogen receptor (ER) and human epidermal growth factor receptor 2 (HER2) status evaluation plays a vital role in determining treatment options for patients with breast cancer. ER-positive tumors are known to be driven by estrogen, making endocrine therapies such as selective estrogen receptor modulators (SERMs) or aromatase inhibitors (AIs) effective treatment options. On the other hand, HER2-positive tumors exhibit an overexpression or amplification of the HER2 receptor [7,8].

Tumor biology is known to evolve over time, and changes in ER and HER2 status may occur. Acquisition of resistance to endocrine therapies or development of HER2 amplification can impact treatment responses [9]. Therefore, reassessing ER and HER2 status provides crucial information about the current state of the disease and its potential response to targeted therapies. NICE acknowledges the exception when no tissue is available and further biopsy is not feasible or declined.

The decision to reassess ER and HER2 status in recurrent breast carcinoma should be guided by the potential impact on treatment management. If the reassessment results have the potential to alter the choice of therapy, it becomes imperative to reconsider the receptor status.

For instance, if a tumor that was initially ER-positive becomes ER-negative upon recurrence, it would indicate a potential loss of response to endocrine therapies and necessitate a shift toward alternative treatment strategies. Similarly, the conversion from HER2-negative to HER2-positive status might prompt the consideration of HER2-targeted agents, leading to a change in therapeutic management [10].

On the other hand, if reassessing receptor status does not offer any immediate clinical implications, it may be unnecessary, as it could increase patient burden and cost, and potentially delay treatment initiation. In such cases, the primary focus should be on maximizing disease control, managing symptoms, and improving overall quality of life.

Endocrine therapy targets the ER pathway, which plays a crucial role in the progression of ER-positive breast cancer. By inhibiting estrogen signaling, endocrine therapy effectively halts tumor growth, reduces the risk of disease recurrence, and improves overall survival rates. It offers numerous benefits, including oral administration, favorable side effect profiles compared to chemotherapy, and long-term disease control.

Clinical studies and real-world evidence have consistently shown the efficacy of endocrine therapy in the treatment of ER-positive advanced breast cancer. In many cases, endocrine therapy has demonstrated comparable or superior efficacy to chemotherapy as a first-line treatment [11]. For patients with non-imminently life-threatening or non-visceral organ involvement, endocrine therapy can provide meaningful disease control and symptom relief, contributing to a better quality of life.

In cases where ER-positive advanced breast cancer poses an imminent life-threatening risk or involves significant visceral organ involvement, a more aggressive approach may be necessary. Patients in such situations require prompt relief of symptoms and disease control. Chemotherapy, with its broader cytotoxic effects, can offer faster response rates and shrink tumors more rapidly. However, it is crucial to ensure that patients understand and accept the potential toxicities associated with chemotherapy.

For patients who have already initiated chemotherapy as their first-line treatment for ER-positive advanced breast cancer, transitioning to endocrine therapy upon completion of chemotherapy is recommended. Chemotherapy often serves as an initial strategy to achieve rapid tumor reduction and symptom relief. Following this, endocrine therapy can be introduced to provide long-term disease management, reduce the risk of recurrence, and minimize the cumulative toxicity associated with continuous chemotherapy. By tailoring treatment strategies to individual patient needs, healthcare providers can optimize outcomes and ensure the best possible care for patients with metastatic breast cancer.

Bone metastases in patients with advanced breast carcinoma often lead to severe pain and reduced quality of life. External beam radiotherapy (EBRT) is a well-established treatment modality used to alleviate pain and control bone metastases [12]. In recent years, a growing body of evidence has suggested that delivering EBRT in a single fraction of 8Gy can be highly effective and advantageous for patients in terms of both pain management and convenience.

A single fraction of 8Gy provides comparable or even better pain relief compared to conventionally fractionated radiotherapy. The condensed treatment schedule allows for a rapid response, with significant pain reduction occurring within days of treatment initiation [13]. This approach is particularly beneficial for patients who require immediate pain relief or have limited life expectancy. It also significantly reduces the number of treatment sessions required. Conventional radiotherapy often involves multiple fractions administered over several weeks. Single-fraction radiotherapy eliminates the logistical challenges and inconvenience associated with frequent hospital visits, making it more patient-friendly. This simplified treatment approach can improve patient compliance and reduce the burden on healthcare resources.

Single-fraction radiotherapy using a dose of 8Gy offers a promising and effective treatment option for patients with advanced breast carcinoma and bone metastases. Its ability to provide superior pain relief, simplified treatment regimen, cost-effectiveness, and comparable outcomes make it an attractive alternative to conventionally fractionated radiotherapy. As the field of radiation oncology continues to evolve, the use of single-fraction radiotherapy is likely to become more prevalent, benefiting patients by improving their quality of life and overall treatment experience.

Axillary lymph node dissection (ALND) is commonly performed in patients with breast cancer to determine the spread of cancer cells and to guide further treatment decisions. While ALND is crucial for cancer management, it can lead to a distressing side effect known as secondary lymphedema. It is characterized by the accumulation of lymphatic fluid in the affected limb, resulting in swelling, discomfort, and reduced function [14].

Exercise is increasingly recognized as a key component of secondary lymphedema management. Contrary to earlier beliefs that physical activity might worsen the condition, numerous studies have shown that properly prescribed exercise can have significant benefits [15]. Exercise helps stimulate the lymphatic system, promoting the drainage of excess fluid and reducing limb swelling. It also enhances muscle strength, joint flexibility, and overall physical fitness, which are crucial for minimizing functional limitations caused by lymphedema.

The most effective exercise programs for secondary lymphedema typically involve a combination of early aerobic exercises, resistance training, flexibility exercises, and manual lymphatic drainage techniques. Aerobic exercises like walking and cycling increase blood circulation and lymphatic flow, facilitating the removal of excess fluid, and should be encouraged early on after surgery, as they are very effective [16].

Advanced-stage breast carcinoma with brain metastasis remains a significant clinical challenge. In cases where surgical intervention is not appropriate or feasible, whole-brain radiotherapy (WBRT) has emerged as a potential therapeutic modality [17]. However, it is crucial to carefully consider patient prognosis before recommending WBRT as a treatment option [18].

WBRT involves the delivery of radiation to the entire brain, aiming to target and control the growth of cancer cells that have spread to the brain. It is a non-invasive treatment option that can provide palliative benefits by reducing symptoms associated with brain metastases, such as headaches, seizures, and neurological deficits. Additionally, WBRT has the advantage of treating both visible and microscopic tumors in the brain, potentially improving overall disease control.

When considering WBRT for advanced-stage breast carcinoma patients, it is essential to assess the patient's prognosis carefully. This evaluation should include factors such as the extent of systemic disease, performance status, comorbidities, and the presence of extracranial metastases. Patients with a favorable prognosis, who are expected to have a reasonable life expectancy and good performance status, are more likely to benefit from WBRT. These patients may experience symptom relief, preservation or improvement of neurologic function, and potentially an extended survival period.

## Conclusions

By evaluating the advanced breast carcinoma care provided in Pakistan's largest cancer hospital against the NICE guidelines, this research has contributed to the understanding of the current state of breast cancer management in the local context. The findings serve as a reference point for healthcare professionals, policymakers, and researchers, providing valuable insights into the strengths and areas for improvement within the healthcare system. it is essential to continue conducting clinical audits and benchmarking efforts, using international guidelines as a compass for excellence. This ongoing evaluation will contribute to the continuous enhancement of breast cancer care and overall healthcare standards in Pakistan. It is our hope that this audit will inspire further investigations, leading to advancements in treatment approaches, better patient outcomes, and ultimately, improved healthcare practices nationwide.

This clinical audit has its limitations because this was done outside of NHS and in a hospital where hospital-wide local guidelines are followed instead of NICE guidelines.

## Appendices

**Table 2:** Audit standards Audit standards are taken from the NICE guidelines on advanced breast cancer: diagnosis and treatment [18]. NICE: National Institute for Health and Care Excellence.

Sr. #	Audit standards		Guidance reference	Exceptions	Definitions	Questions
	Diagnosis and assessment					
1.	Assess the presence and extent of visceral metastases using a combination of plain radiography, ultrasound, computed tomography (CT) scans, and magnetic resonance imaging (MRI).		1.1.1	None	None	1
2.	Positron emission tomography fused with computed tomography (PET-CT) should only be used to make a new diagnosis of metastases for patients with breast cancer whose imaging is suspicious but not diagnostic of metastatic disease.		1.1.5	None	None	2&3
3.	On recurrence, consider reassessing estrogen receptor (ER) and human epidermal growth factor 2 receptor (HER2) status if a change in receptor status will lead to a change in management.		1.1.6	1. When no tissue is available and further biopsy is not feasible or declined	None	4, 5, 6, 7, & 8
	Disease monitoring					
4.	Do not use PET-CT to monitor advanced breast cancer.		1.1.8	None	None	10 & 11
	Systemic disease-modifying therapy					
5.	Offer endocrine therapy as first-line treatment for the majority of patients with ER-positive advanced breast cancer.		1.3.1	2. The patient has ER-positive advanced breast cancer, which is imminently life-threatening or requires early relief of symptoms because of significant visceral organ involvement, provided they understand and are prepared to accept toxicity. 3. The patient is already being treated with chemotherapy	Chemotherapy should be offered as first-line treatment for patients with ER-positive advanced breast cancer whose disease is imminently life-threatening or requires early relief of symptoms because of significant visceral organ involvement, provided they understand and are prepared to accept toxicity. Patients with ER-positive advanced breast cancer who have been treated with chemotherapy as their first-line treatment should be offered endocrine therapy following the completion of chemotherapy	4, 7, & 9
	Managing complications					
6.	Where external beam radiotherapy is used to treat patients with bone metastases and pain, this should be done in a single fraction of 8Gy		1.5.16	4. Where treatment of other potential or current bone complications requires longer fractionation	None	12, 13, 14, & 15
7.	Discuss with people who have or who are at risk of breast cancer-related lymphedema that exercise may improve their quality of life.		1.5.2	None	None	16
8.	Offer whole brain radiotherapy to patients for whom surgery is not appropriate unless they have a very poor prognosis.		1.5.19	None	None	17 & 18
	Uncontrolled local disease					
9.	A breast cancer multidisciplinary team should assess all patients presenting with uncontrolled local disease and discuss the therapeutic options for controlling the disease and relieving symptoms.		1.5.11	None	The multidisciplinary team should discuss therapeutic options for controlling the disease and relieving symptoms	19

**Table 3:** Form used for data collection Audit standards are taken from the NICE guidelines on advanced breast cancer: diagnosis and treatment [18]. NICE: National Institute for Health and Care Excellence; TAP: thorax, abdomen, and pelvis.

AUDIT ID #	MR #	Name:	Age/sex:

No.	Questions	Yes	No	Exceptions*/NA
1.	Was adequate metastatic workup done for staging? CT-TAP, bone scan?			
2.	Was the patient’s imaging suspicious but not diagnostic of metastatic disease?			
3.	Was PET-CT used to make a new diagnosis of metastases?			
4.	Was the receptor status assessed at the time of initial diagnosis?			

5. Was there any local re-occurrence, mention date if any?			
6. Was the receptor status assessed at the time of disease recurrence?	Yes	No	Exceptions*

No.	Question	Estrogen (ER)	Progesterone (PR)	Human epidermal growth factor receptor 2 (HER2)	Exceptions*/NA
7.	What was the patient’s initial receptor status?				
8.	What was the patient’s receptor status on recurrence?				

No.	Question	Yes	No	Exceptions*/NA
9.	If the patient was ER-positive, were they offered endocrine therapy as the first-line treatment?			
10.	Was PET-CT done more than once?			
11.	Was PET-CT done for re-staging scans?			
12.	Was external beam radiotherapy offered to patients with bone metastasis?			
13.	Were there other potential or current bone complications?			
14.	Please state doses if bone radiotherapy was done.			
15.	Number of fractions			
16.	Patients with a risk of lymphedema (after axillary clearance surgery) seen by a physiotherapist?			
17.	In the case of brain metastases, was the brain metastases pathway followed?			
18.	Brain radiotherapy?			
19.	Was the patient assessed by a multidisciplinary team?			
